# The Chemistry of Synthetic Drugs

**Published:** 1911-12

**Authors:** 


**The Chemistry of Synthetic Drugs.** By Percy May, B.Sc. Pp. xiii, 229. London: Longmans, Green & Co. 1911.

In this book a considerable number of the synthetic drugs and also of other bodies used in medicine, such as the alkaloids, glucosides and terpenes, are dealt with almost entirely from the chemical point of view. As the author acknowledges in his preface, the book owes a considerable debt to Frænkel's *Artzneimittelsynthese;* in fact, a large number of the chapters are modelled on that work, and the greater part of the references seem to be drawn from the same source. Certain chapters, such as those relating to the adrenaline bodies and the arsenic and antimony compounds, are derived from sources more recent than Frænkel's second edition, which was published in 1906, and the account of the morphine group of alkaloids has also been brought up to date. On the other hand the chapter on glucosides, camphor and the glycerophosphates might, we think, have been expanded with advantage; the account of the glucosides especially being meagre, as is also the case in Frænkel's book, where only two pages are devoted to this group of bodies.

Mr. May begins his book with a glossary of terms, the exact object of which is not quite apparent. It seems to us obvious that those who require to be told the meaning of the word "styptic" will not be in a position to read with advantage a book dealing with the advanced chemistry of drugs; nor will those whose knowledge of chemistry does not extend to the comprehension of the term "alkyl" be able to follow the complicated structural formulæ which occur on almost every page with either facility or profit.

The arrangement and contents of the first three chapters do not differ from those in other works on the same subjects, and include the action of synthetic compounds, the effect of various radicles and elements, and the chemical changes occurring in drugs when introduced into the organism. The detailed account of various bodies which follows suffers, we think, from the fact that it is written too exclusively on chemical lines; the author gives but little pharmacological detail, and is evidently not in a position to deal with the practical and clinical values of the numerous remedies enumerated. Thus cosaprin is merely stated to be "inert," exalgin "toxic," euphorin "irregular in its antipyretic effect." Drugs which have obtained some vogue in practice, such as cryogenia and maretin, are not mentioned, so far as we have been able to ascertain. Frænkel, it is true, devotes only a line or two to each, but there is a considerable literature available on both elsewhere. Veronal sodium (medicinal) is mentioned as being valuable on account of its solubility, although from the practical standpoint this statement requires considerable modification.

No mention is made, again, of urea and quinine hydrochloride, which has recently come into prominence as a local anæsthetic, though several of the older quinine compounds are briefly dealt with.

The loose way in which the author deals with pharmacological points is illustrated on page 51, where with regard to phenacetin he states that "its antipyretic action is due to increased heat loss from the surface of the body"—a remark equally applicable to the action of a cold bath—and further that the analgesic action "is *mainly* due to its action on the sensory tracts of the cord." The italics are ours.

In the short account (on page 190) of the theories regarding the physiological action of organic arsenic compounds of the atoxyl type, the important contribution of Boeinl and Nierenstein to this subject is not mentioned.

From the pharmacological point of view, therefore, the book appears to us deficient, but from the chemical standpoint we regard it as a creditable attempt to bring together a selection of the material contained in Frænkel's large work, with other more recent synthetic processes in pharmaceutical chemistry.

We have detected remarkably few typographical mistakes, which in a book so full of formulæ is evidence of very careful proof reading. On page 49 "absorption" should be "adsorption"; on page 59 in the formula for sulphonal $H_2$ should stand for H; on page 128 "fushion" should be "fusion"; on page 178 potassium "iodide" should be "bromide"; on page 200 "tarter emetic" should be "tartar emetic." We also think that it is a mistake to print the same formula in a different way on different pages as is done for pyrazolon derivations on page 20 and page 65, and for ecgonine on page 88 and page 91. On page 125 stypticine should be described as the hydrochloric acid salt of di-hydro-isoquinoline.

